# Identification of a druggable binding pocket in the spike protein reveals a key site for existing drugs potentially capable of combating Covid-19 infectivity

**DOI:** 10.1186/s12860-020-00294-x

**Published:** 2020-07-01

**Authors:** Elliot D. Drew, Robert W. Janes

**Affiliations:** School of Biological and Chemical Sciences, Queen Mary University of London, Mile End Road, London, E1 4NS UK

## Abstract

**Background:**

Following the recent outbreak of the new coronavirus pandemic (Covid-19), the rapid determination of the structure of the homo-trimeric spike glycoprotein has prompted the study reported here. The aims were to identify potential “druggable” binding pockets in the protein and, if located, to virtual screen pharmaceutical agents currently in use for predicted affinity to these pockets which might be useful to restrict, reduce, or inhibit the infectivity of the virion.

**Results:**

Our analyses of this structure have revealed a key potentially druggable pocket where it might be viable to bind pharmaceutical agents to inhibit its ability to infect human cells. This pocket is found at the inter-chain interface that exists between two domains prior to the virion binding to human Angiotensin Converting Enzyme 2 (ACE2) protein. One of these domains is the highly mobile receptor binding domain, which must move into position to interact with ACE2, which is an essential feature for viral entry to the host cell. Virtual screening with a library of purchasable drug molecules has identified pharmaceuticals currently in use as prescription and over the counter medications that, in silico, readily bind into this pocket.

**Conclusions:**

This study highlights possible drugs already in use as pharmaceuticals that may act as agents to interfere with the movements of the domains within this protein essential for the infectivity processes and hence might slow, or even halt, the infection of host cells by this new coronavirus. As these are existing pharmaceuticals already approved for use in humans, this knowledge could accelerate their roll-out, through repurposing, for affected individuals and help guide the efforts of other researchers in finding effective treatments for the disease.

## Background

Coronaviruses are a family of envelope viruses which are hosted primarily by mammals and by birds. Their general structure comprises of a single-stranded positive sense RNA genome which creates four viral proteins, the S (spike), N (nucleocapsid), M (membrane) and E (envelope) proteins. Each has at least one key role: M and E make up the primary protein components of the viral envelope defining its shape and having a major role in virus propagation, respectively. N is involved in stabilising the nucleocapsid binding directly to the RNA viral material. The spike protein (S), is pivotal to viral infection as it is this that binds to receptors on the host cells enabling subsequent fusion between the host and viral membranes such that the interior RNA material can then invade the host cell [1]. The S protein is comprised of three identical polypeptide subunits arranged as a trimer structure. It is this protein that gives the virus its name as the end of the spike has the appearance of a small “crown” in shape. In this end region is a mobile domain which moves from an inaccessible (down) state to an accessible (up) state which makes it available for interaction with Angiotensin Converting Enzyme 2 (ACE2), the transmembrane protein through which coronavirus infection usually proceeds. It is the spike protein therefore, that is critical for infectivity and in this regard it can be considered as the optimal target for vaccine and drug intervention, being the most prominent on the surface of the virion.

In recent years these types of viruses have posed a major threat due to their ability to cross the species barrier, causing infection in the human population from a virus innately from another source, mammal or bird. Such cross-species events have resulted in Severe Acute Respiratory Syndrome (SARS) [2] and Middle Eastern Respiratory Syndrome (MERS) [3] which arose from bats and then jumped to humans from civet and camel, respectively, as intermediaries [4]. Such viruses that arise in this way within the human population are potentially very problematic in that we have no innate antibodies to these virions and so there is always the possibility for severe illness and death to occur as a result. A new cross-species event has recently arisen in Wuhan City in China, now termed Covid-19. The source is still being debated, although it is likely this virus again originated in bats and then crossed to humans probably again using a mammalian intermediary.

The spike (S) protein from the SARS and MERS coronaviruses have been studied in detail and a number of X-ray and cryo-electron microscopy (Cryo-EM) structures have been produced. Gaining detailed information about these structures offers ways both of understanding how the spike protein is used to infect host cells, and of combating this infectivity. To date, two structural models of this new coronavirus spike protein have been produced, one [5] developed using C-I-Tasser [6], the other [7] produced by Swiss Modeller [8] using as templates homologous structures from the SARS and MERS spike proteins. Both models were used for evaluating the possibility of its binding to ACE2, and Zhang et al. [5] also dispelled suggestions of the presence of novel sequence inclusions in this protein.

**Fig. 1:**
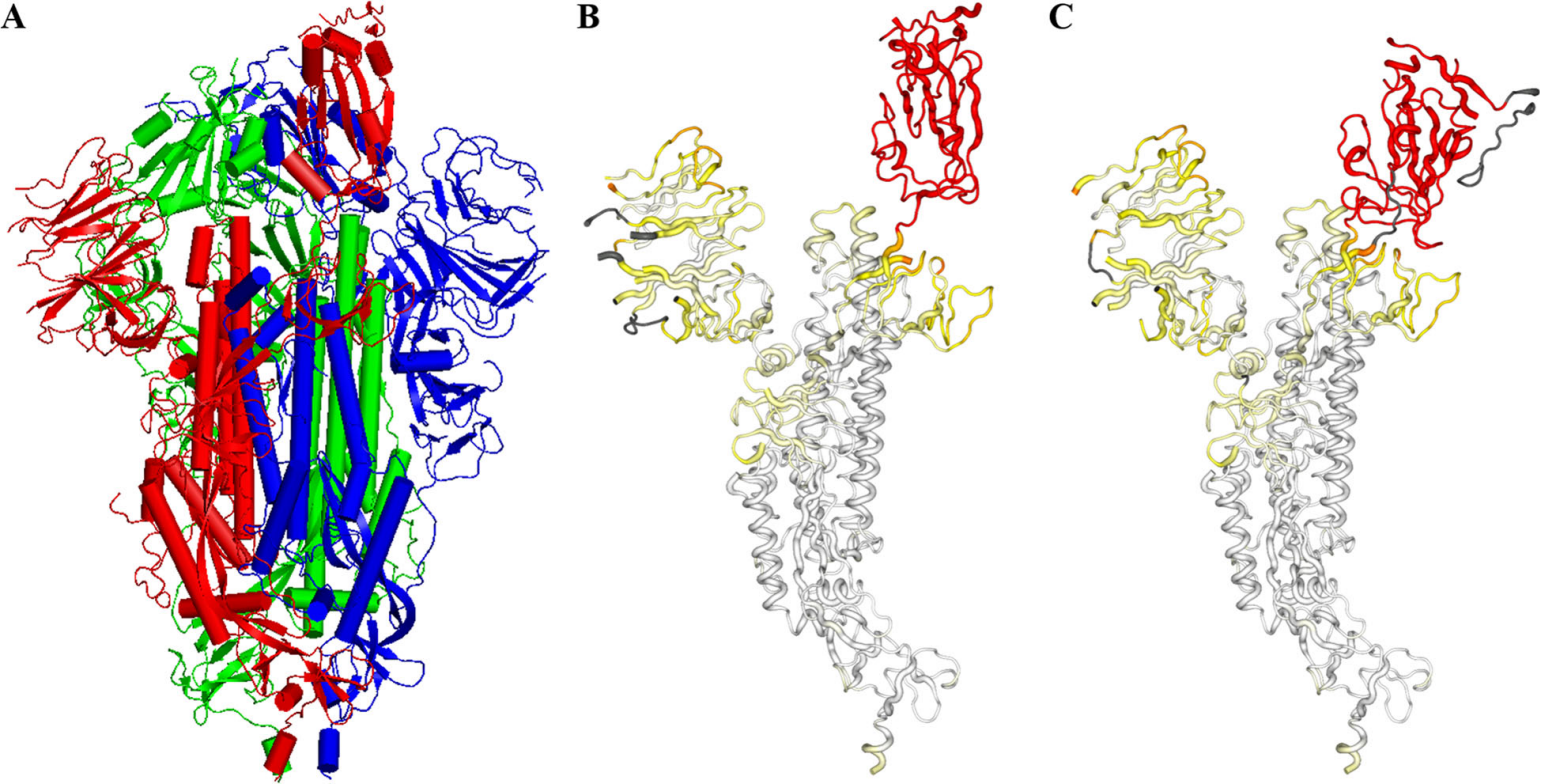
**a** The structure of the Spike (S) protein from Covid-19 (PDB code 6VSB) showing its three subunit conformation. In red is chain A in the “up” position, and chains B and C (blue and green respectively) are in the “down” position (see text for specific details). **b** Chain A in the up position and **c** chain B in the same orientation as A, in the down position. These are coloured according to the structural differences between them where red indicates the most significant of these differences, using 2StrucCompare [10]. Structure coloured grey only exists in chain B

Of key importance here, is the recently reported Cryo-EM structure, solved to 3.5 Å resolution, of the spike protein of Covid-19 [9] and the coordinates for this structure are available under the Protein Data Bank (PDB) code 6VSB; it is this structure that prompted the investigations reported here to look for sites where it might be possible to bind drugs. The structure here is interesting as one subunit chain (A) is in the “up” accessible state, while the other two (B and C) are in the “down” inaccessible state. The structure is shown in Fig. 1 where the differences in conformation of the up and down states are also shown. Figure 1b shows chain A in the up state, whilst Fig. 1c shows the same view of chain B but in the down state; the mobile domain is coloured red in both cases, while structure that is in dark grey only exists in the one chain, B in this case. The protein retains its three fold symmetry over all regions of its structure that do not interact with this mobile domain. The aims were to see if pharmaceutical products currently available and approved for human use might be able to bind to the spike protein with the chance that this might disrupt, stall, or even prevent, the infection process. In order to infect, the spike protein has to be exposed and then become accessible within the host so it can contact the receptors on the host cell. If infectivity were able to be slowed down because of disruption of the process, this would leave the spike protein still exposed but in the inaccessible state and this would enable the host to register the virion as being foreign and so antibodies would be raised against it.

## Results

### Pocket identification

**Fig. 2:**
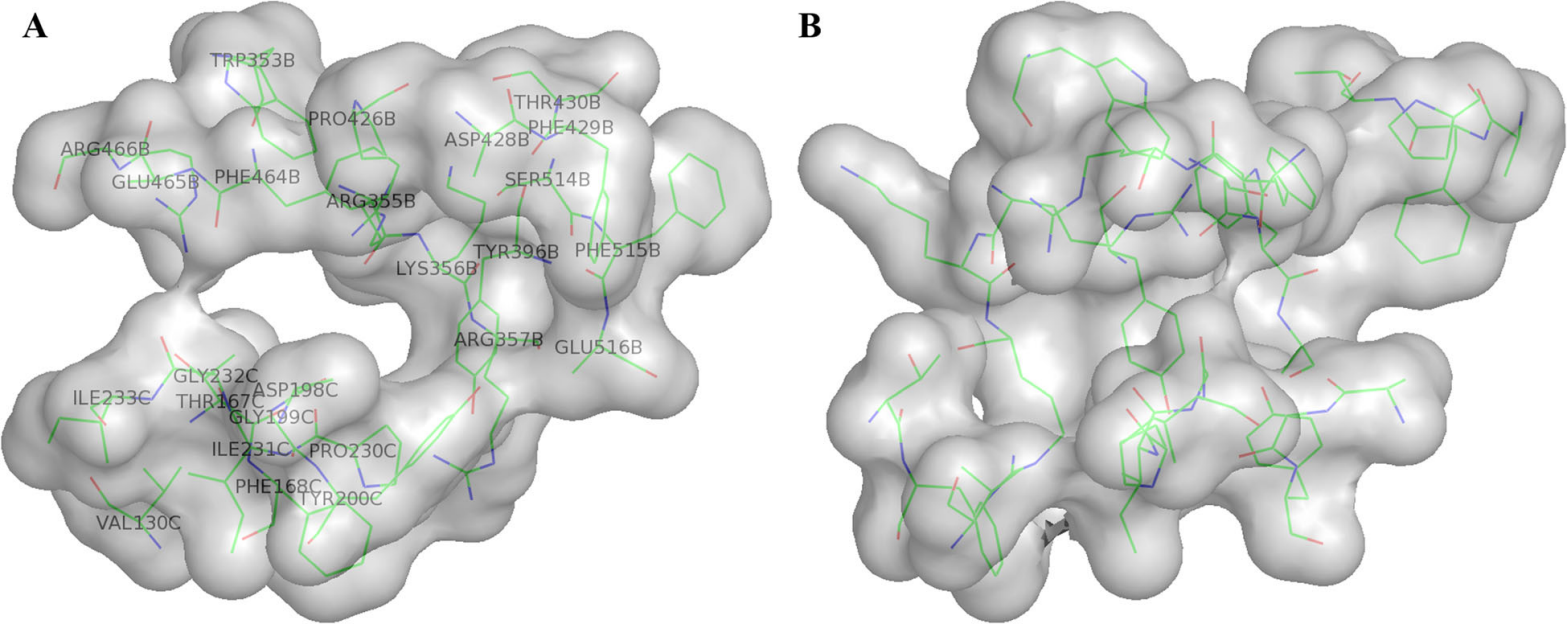
The key pocket proposed to exist in the Cro-EM spike protein structure from Covid-19 from the results of the DoGSiteScorer server. The site is adjacent to where major movements occur when the virus changes conformation on encountering the host cell receptor, preparing it for infection. **a.** Depicts the residues lining the pocket, name and sequence number, along the “axis” of the pocket. **b.** 90^O^ rotation about the viewed vertical axis from the view in A perpendicular to the “axis” of the pocket. This view is the one used to show the chosen poses in Fig. 5

**Fig. 3:**
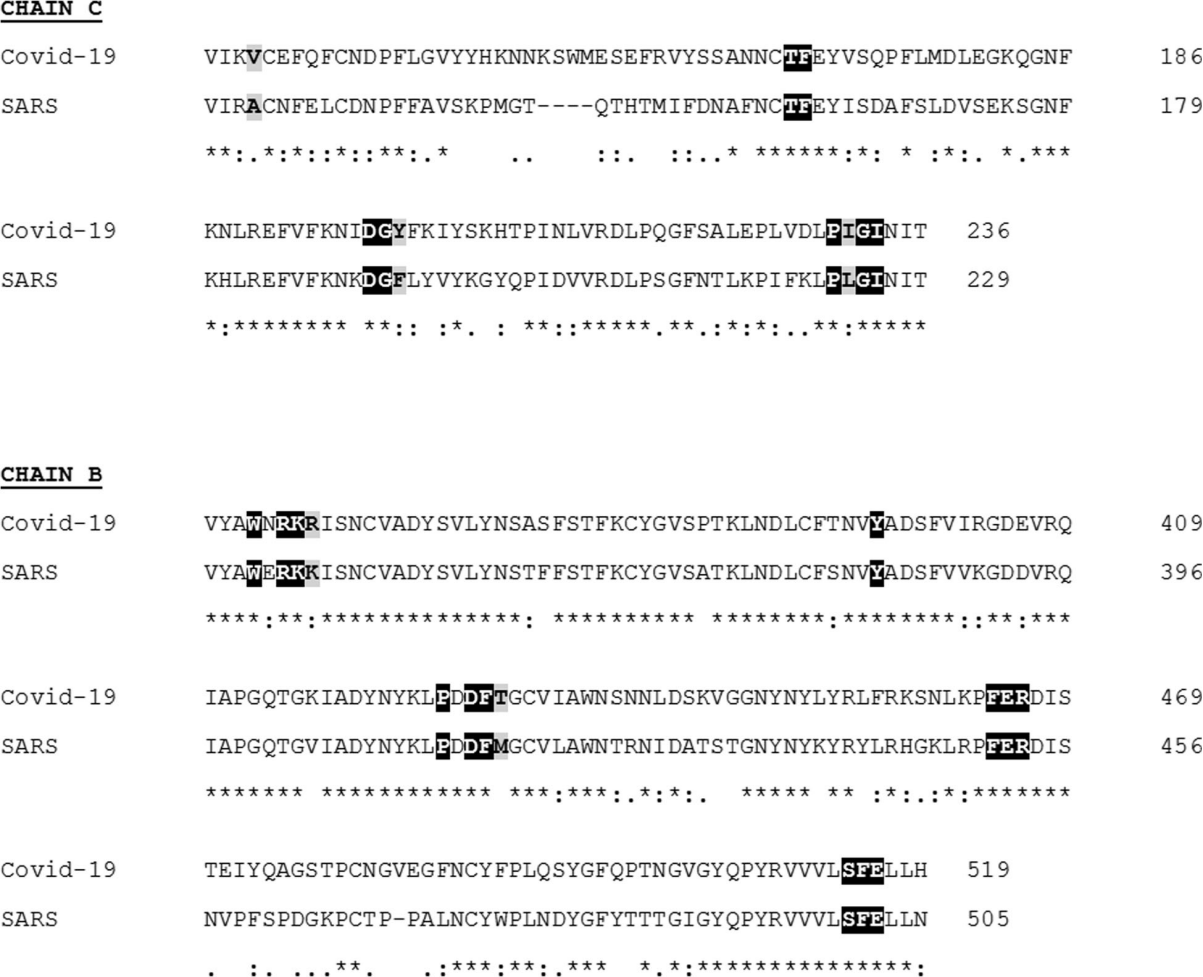
Sequence homology between the residues of the Covid-19 and SARS spike proteins highlighting those residues lining the pocket in the Covid-19 structure. Those residues that are identical are in white lettering on black background (20 of 25 and indicated with an asterisk), whilst the remaining that are conservatively replaced (4 of 25 indicated by a colon or full stop) are in bold black lettering on a grey background

**Fig. 4:**
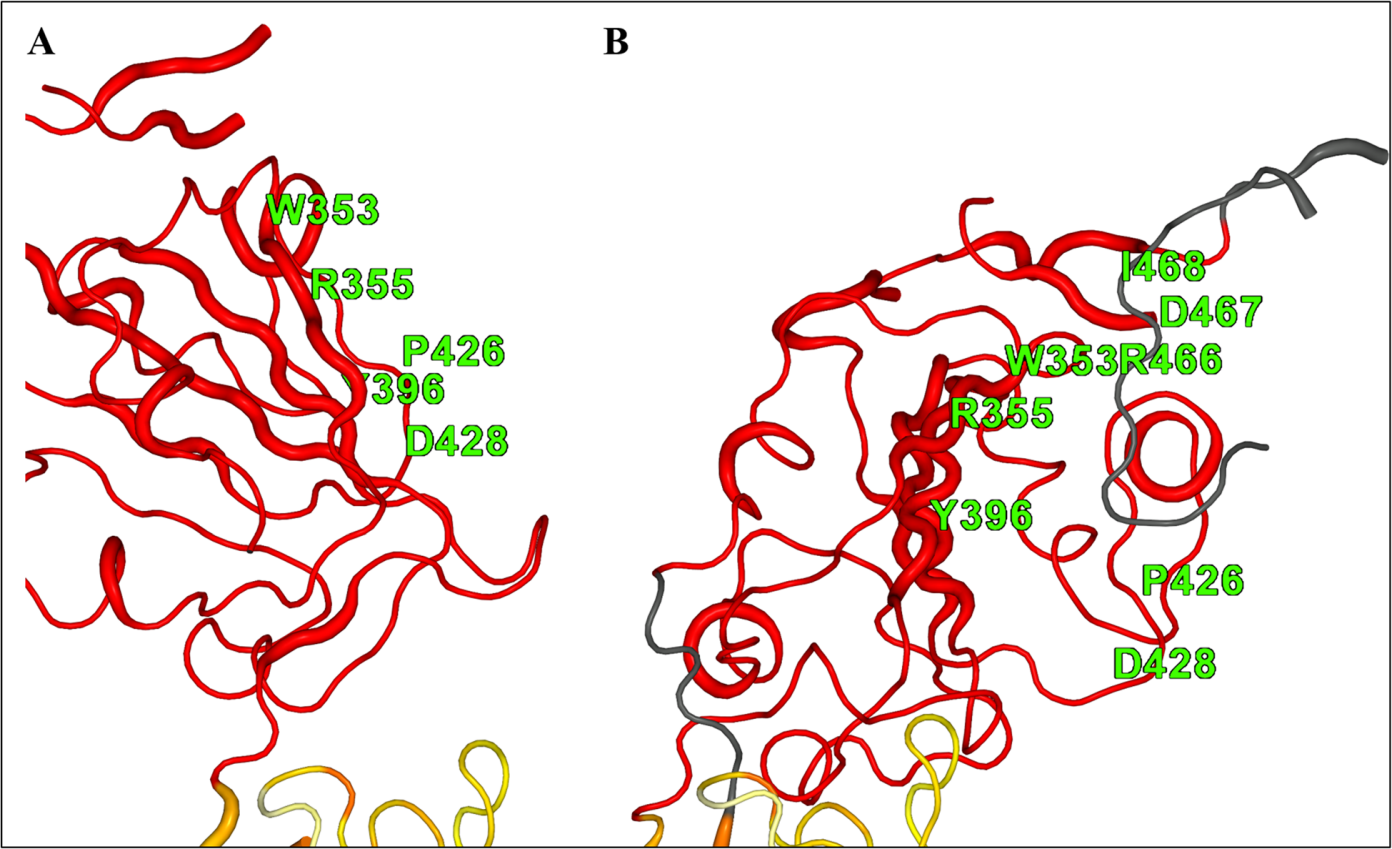
Images showing the differences between paired C-alpha residues from chain A and chain B of the spike protein coloured according to their extent of difference using 2StrucCompare [10]. Residues coloured in red indicate differences in paired positions greater than 5 Å from each other between the two chains. **a.** Shows the shape and position of chain A in the “up” position. **b**. Shows the same orientation for chain B in the “down” position. **S**ome residues are labelled to indicate approximately where the pocket residues are in the two positions of these chains, and in B three of these residues lie on the strand coloured dark grey indicating that this is not seen in chain A

Results from the DoGSiteScorer server [11] identified a total of 106 possible pockets within the Cryo-EM structure, with druggability scores ranging from 0.125 to 0.849 over a scoring range between 0 and 1, where 1 would be a perfect druggable site. Of these 106 possible sites, 79 had a druggability score less than 0.7, and a further 7 did not have a three-fold symmetry where it should be expected, leaving 20 remaining sites. Removing pockets with a small overall volume (less than 500 Å^3^) as these were considered as unlikely to be successfully druggable [11], left 12 potential candidate pockets. Analysis of these revealed an ~ 800 Å^3^ pocket with a high druggability score of 0.79 which was in the 90th percentile of those identified (Table 1 and Fig. 2), and its location, between the mobile domain (in the down position) of one subunit and a second subunit, suggested it could be of potential interest regarding infectivity. Comparison between the residues of Covid-19 lining this pocket and the matched SARS spike protein residues is given in Fig. 3. Figure 4 shows the structural comparisons between chain A and B in the up and down states with selected pocket residues identified to highlight the positional changes.

**Table 1:** Residues lining the druggable pocket identified from the DoGSiteScorer server

Chain B Residues	Chain C Residues
Trp 353	Val 130
Arg 355	Thr 167
Lys 356	Phe 168
Arg 357	Asp 198
Tyr 396	Gly 199
Pro 426	Tyr 200
Asp 428	Pro 230
Phe 429	Ile 231
Thr 430	Gly 232
Phe 464	Ile 233
Glu 465	
Arg 466	
Ser 514	
Phe 515	
Glu 516

### Docking methods

The results from the three methods, Autodock vina [12], Smina [13] and Ledock [14], were used to generate a list of 4358 poses of commercially-available drugs capable of binding into this Covid-19 spike protein pocket. None of the pharmaceutical agents that were successfully docked into the site displayed any steric hindrance within the site and all of these had favourable empirically calculated binding energies.

### Analysis of the compound rankings

From the extensive spreadsheet of data, obtained from the results of the Autodock vina, Smina and Ledock methods, together with related information associated with the drugs within the list (supplied as Supplementary Information), analyses were undertaken to establish the presence of any correlations between, or statistical significance within these results to aid further investigation. Correlations were obtained between various properties, looking for the differences/similarities between them over the whole data set of results. Specifically, the chemical and geometrical properties of the top 150 conformers, as ranked by ComboPC score, were compared against the full dataset to see if any pattern emerged concerning the top performing compounds. To assess the significance of any difference found, a t-test was employed and a ratio of the top 150 set average versus the whole set average was obtained to assess the direction and magnitude of any difference.

### Structure families

**Fig. 5:**
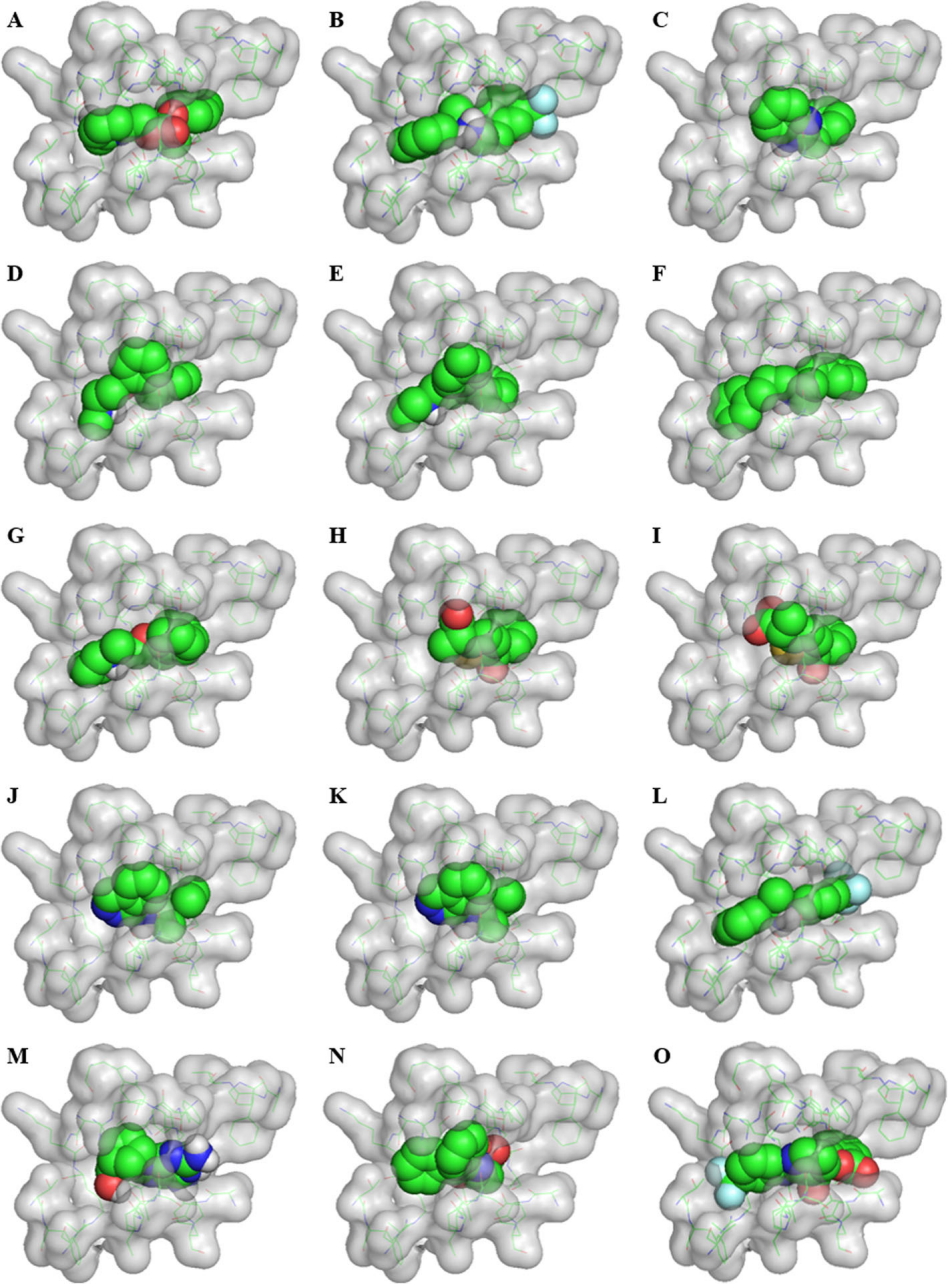
Fifteen poses (using Ledock poses) from the top 100 pharmaceutical agents listed in the data ordered by ComboPC score. Fourteen of these are actively-used pharmaceutical compounds and the last retains the potential to be active and is given because of its pharmaceutical activity towards cystic fibrosis, chronic obstructive pulmonary disease (COPD), and asthma. These are: **a**. Pirifibrate **b**. Cinacalcet **c**. Bendazol **d**. Phenyltoloxamine **e**. Triprolidine **f**. Alverine **g**. Benproperine **h**. and **i**. Tiaprofenic acid **j**. and **k**. Budralazine **l**. Cinacalcet **m**. Abacavir **n**. Oxaprozin **o**. Talniflumate

**Fig. 6:**
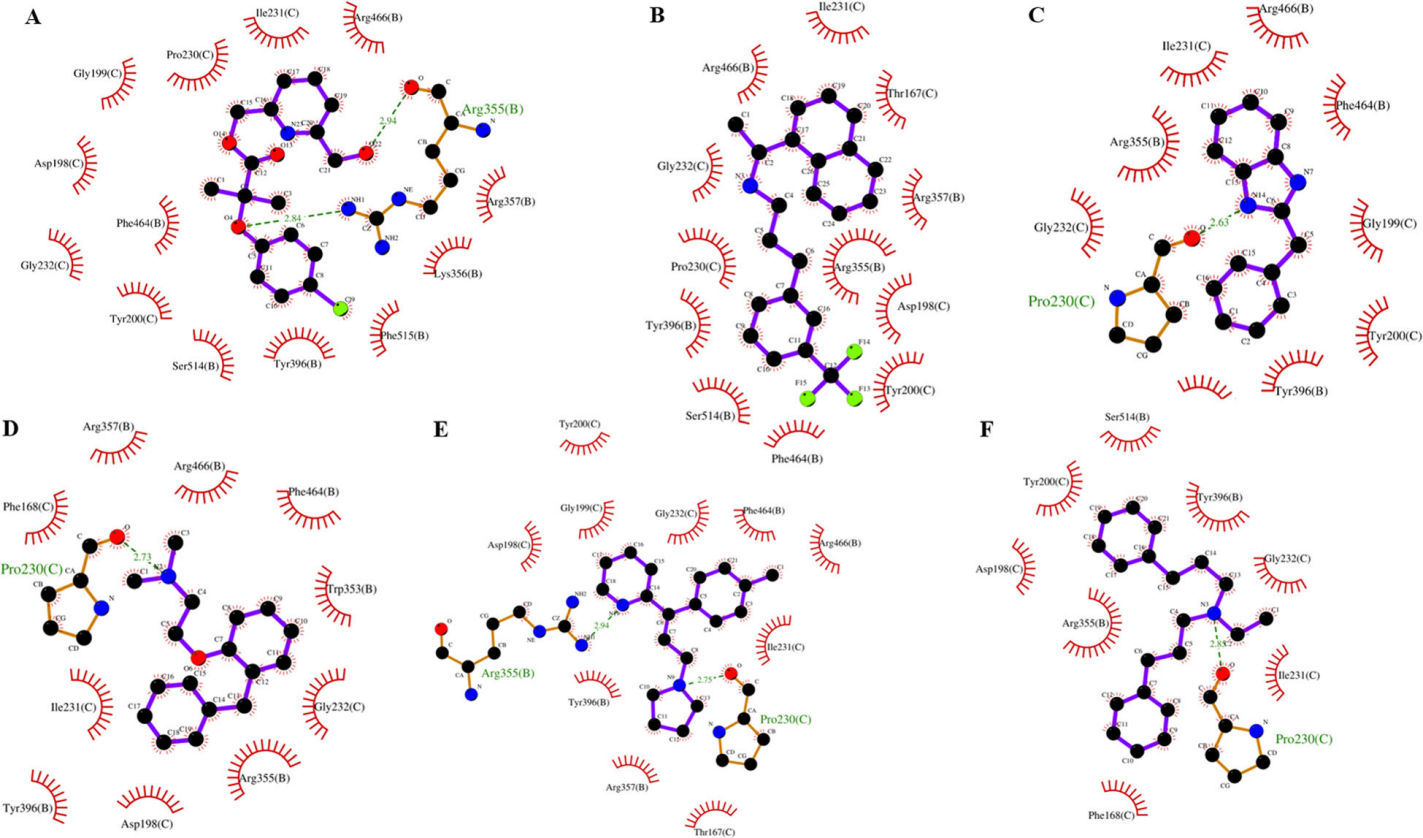
Selected Ligplot [15] diagrams from the fifteen poses in Fig. 5 (using Ledock poses). These figures indicate the interactions between the drug (the ligand, displayed in ball and stick format) and the surrounding pocket residues. Hydrogen bonds are indicated by a dashed line between the drug ligand atom and the atom of the residue lining the pocket which is also displayed in ball and stick in these instances. Van der Waals contacts are indicated by “fans” of lines around each residue directed towards the drug which also have reciprocal fans directed towards each of the residues lining the pocket with which it is interacting. The plots are for the drugs: **a**. Pirifibrate **b**. Cinacalcet **c**. Bendazol **d**. Phenyltoloxamine **e**. Triprolidine **f**. Alverine

Table 2 lists the top 100 drug poses ordered by the Combo percentile score. In this table 14 drugs are actively used for treatment and one further drug is highlighted, as it might be available, which is used in the treatment of various pulmonary diseases. These 15 molecules are shown in their poses binding into the pocket in Fig. 5. Six of these are shown in Fig. 6 as selected examples of how these drugs interact with the lining pocket residues. The figures are using the Ledock poses in both cases.

**Table 2:** A list of the top 100 poses of drugs ranked according to their Combo percentile score. The table consists of the Drug name, Pharmaceutical Role, Ledock percentile score (LedockPC), Autodock vina percentile score (VinaPC), avRMSD percentile score (RMSDPC) and overall Combo percentile score (ComboPC). Those structures indicated in bold have poses in Fig. 6. All except Talniflumate are active pharmaceuticals, while this compound can still be active, and is highlighted because of its drug action

Drug name	Pharmaceutical Role^c^	LedockPC	VinaPC	RMSDPC	ComboPC
Irolapride^a^	Antidepressant	0.001	0.004	0.053	0.02775
**Pirifibrate**	**Antilipidemic**	0.065	0.031	0.015	0.0315
Etoloxamine^a^	Antihistamine	0.054	0.101	0.008	0.04275
Timoprazole^a^	Proton pump inhibitor	0.04	0.129	0.008	0.04625
Timoprazole^a^	Proton pump inhibitor	0.048	0.129	0.005	0.04675
Benfluorex^a^	Withdrawn	0.02	0.004	0.084	0.048
Vorozole^a^	Breast cancer	0.044	0.045	0.054	0.04925
Nicotredole^a^	Anti-inflammatory, analgesic	0.056	0.045	0.056	0.05325
Nolinium^a^	Antispasmodic	0.046	0.031	0.069	0.05375
**Talniflumate**^a^	**Cystic fibrosis, COPD, asthma**	0.021	0.012	0.095	0.05575
Veliparib^a^	Anti-cancer	0.07	0.024	0.066	0.0565
Timoprazole^a^	Proton pump inhibitor	0.018	0.061	0.078	0.05875
Oxifungin^a^	Antifungal	0.043	0.08	0.057	0.05925
Fenfluthrin^a^	Insecticide	0.098	0.045	0.054	0.06275
Piketoprofen^b^	Topical anti-inflammatory cream	0.101	0.005	0.075	0.064
Nafazatrom^a^	Antithrombotic	0.134	0.031	0.054	0.06825
Irolapride^a^	Antidepressant	0.004	0.08	0.096	0.069
Etacepride^a^	Neuroleptic, antiemetic	0.019	0.158	0.05	0.06925
Timoprazole^a^	Proton pump inhibitor	0.021	0.061	0.098	0.0695
Irolapride^a^	Antidepressant	0.002	0.08	0.1	0.0705
**Cinacalcet**	**Hyperparathyroidism**	0.169	0.001	0.058	0.0715
Mitoflaxone^a^	Antitumor	0.138	0.031	0.06	0.07225
**Bendazol**	**Vasodilator (Russia)**	0.225	0.045	0.01	0.0725
**Phenyltoloxamine**	**Antihistamine**	0.096	0.194	0.002	0.0735
Eprovafen^a^	Anti-inflammatory	0.067	0.129	0.052	0.075
Irolapride^a^	Antidepressant	0.055	0.158	0.046	0.07625
Losmiprofen^a^	Anti-inflammatory, analgesic	0.134	0.158	0.008	0.077
Benfluorex^a^	Withdrawn	0.008	0.004	0.151	0.0785
Rolodine^a^	Muscle relaxant	0.079	0.08	0.079	0.07925
Dextrofemine^a^	Antispasmodic	0.184	0.061	0.04	0.08125
Fenfluthrin^a^	Insecticide	0.109	0.045	0.087	0.082
**Triprolidine**	**Coughs, upper respiratory**	0.026	0.101	0.103	0.08325
Losmiprofen^a^	Anti-inflammatory, analgesic	0.141	0.129	0.034	0.0845
Rolodine^a^	Muscle relaxant	0.002	0.129	0.105	0.08525
Triafungin^a^	Antifungal	0.281	0.024	0.025	0.08875
Losmiprofen^a^	Anti-inflammatory, analgesic	0.134	0.158	0.033	0.0895
Piketoprofen^b^	Topical anti-inflammatory cream	0.053	0.001	0.154	0.0905
Chlormidazole^a^	Antifungal	0.221	0.101	0.027	0.094
Domoxin^a^	Antithrombotic	0.109	0.194	0.038	0.09475
Nolinium^a^	Antispasmodic	0.052	0.031	0.148	0.09475
Timoprazole^a^	Proton pump inhibitor	0.03	0.268	0.046	0.0975
Diflumidone^a^	Anti-inflammatory	0.043	0.016	0.167	0.09825
Pranosal^a^	Analgesic, anti-inflammatory	0.065	0.194	0.068	0.09875
Tolpentamide^a^	Hypoglycemia	0.052	0.061	0.142	0.09925
Nafazatrom^a^	Antithrombotic	0.243	0.031	0.062	0.0995
Benfluorex^a^	Withdrawn	0.079	0.008	0.156	0.09975
Risarestat^a^	Cornea eye treatment	0.035	0.229	0.07	0.101
Nafimidone^a^	Anticonvulsant	0.165	0.031	0.11	0.104
Butanixin^a^	Analgesic, anti-inflammatory	0.081	0.08	0.128	0.10425
Chlormidazole^a^	Antifungal	0.225	0.101	0.048	0.1055
Vorozole^a^	Breast cancer	0.056	0.045	0.161	0.10575
Irolapride^a^	Antidepressant	0.003	0.004	0.209	0.10625
Colfenamate^a^	Antipyretic, anti-inflammatory	0.009	0.016	0.207	0.10975
Diprofene^a^	Antispasmodic	0.101	0.158	0.09	0.10975
**Alverine**	**IBS**	0.233	0.101	0.053	0.11
Fendiline^a^	Anti anginal	0.259	0.158	0.014	0.11125
**Benproperine**	**Anti-cough**	0.031	0.045	0.185	0.1115
Nicotredole^a^	Anti-inflammatory, analgesic	0.034	0.045	0.184	0.11175
**Tiaprofenic-acid**	**Anti-inflammatory**	0.269	0.158	0.01	0.11175
**Tiaprofenic-acid**	**Anti-inflammatory**	0.269	0.129	0.025	0.112
Diphenan^a^	Anti-worm	0.269	0.061	0.059	0.112
Enfenamic-acid^a^	Topical anti-inflammatory	0.342	0.101	0.004	0.11275
**Budralazine**	**Vasodilator (Japan)**	0.134	0.313	0.002	0.11275
Picobenzide^a^	Neuroleptic	0.295	0.08	0.04	0.11375
Zomepirac^a^	Withdrawn	0.162	0.158	0.068	0.114
Cinchophen^a^	Withdrawn	0.29	0.129	0.019	0.11425
Nolinium^a^	Antispasmodic	0.087	0.08	0.146	0.11475
Pribecaine^a^	Local anesthetic	0.221	0.194	0.022	0.11475
Tepirindole^a^	Experimental	0.138	0.024	0.149	0.115
Etoloxamine^a^	Antihistamine	0.169	0.101	0.095	0.115
**Budralazine**	**Vasodilator (Japan)**	0.144	0.313	0.002	0.11525
Triafungin^a^	Antifungal	0.403	0.024	0.017	0.11525
Nafazatrom^a^	Antithrombotic	0.176	0.045	0.121	0.11575
Triflocin^a^	Diuretic	0.285	0.158	0.011	0.11625
Tolonidine^a^	Antihypertensive	0.233	0.194	0.024	0.11875
Isaglidole^a^	Antidiabetic	0.144	0.194	0.069	0.119
Iquindamine^a^	Antitussive	0.067	0.268	0.071	0.11925
Kinetin^a^	Cell division (plants)	0.037	0.362	0.039	0.11925
**Cinacalcet**	**Hyperparathyroidism**	0.024	0.001	0.227	0.11975
Bencisteine^a^	Antitussive	0.013	0.229	0.119	0.12
Salazosulfamide^a^	Ankylosing spondylitis	0.002	0.045	0.217	0.12025
Picobenzide^a^	Neuroleptic	0.29	0.08	0.057	0.121
Triflocin^a^	Diuretic	0.239	0.061	0.092	0.121
Timoprazole^a^	Proton pump inhibitor	0.101	0.129	0.128	0.1215
Fenclofenac^a^	Withdrawn	0.169	0.158	0.08	0.12175
Ridogrel^a^	Thrombo-embolism	0.094	0.012	0.191	0.122
Ibuprofen-piconol^b^	Topical anti-inflammatory cream	0.217	0.045	0.114	0.1225
Tecalcet^a^	Hyperparathyroidism	0.123	0.101	0.135	0.1235
Tazadolene^a^	Antidepressant	0.364	0.061	0.035	0.12375
Piridocaine^a^	Anaesthetic	0.081	0.229	0.094	0.1245
Veliparib^a^	Anti-cancer	0.03	0.024	0.222	0.1245
Ibuprofen-piconol^b^	Topical anti-inflammatory cream	0.2	0.101	0.103	0.12675
Furobufen^a^	Anti-inflammatory	0.249	0.194	0.032	0.12675
Prefenamate^a^	Anti-inflammatory	0.008	0.061	0.22	0.12725
Bakeprofen^a^	Analgesic, antipyretic	0.281	0.045	0.092	0.1275
Fenfluthrin^a^	Insecticide	0.372	0.045	0.047	0.12775
Isaglidole^a^	Antidiabetic	0.148	0.194	0.086	0.1285
Clomacran^a^	Withdrawn	0.007	0.061	0.224	0.129
**Abacavir**	**HIV treatment**	0.003	0.268	0.124	0.12975
**Oxaprozin**	**Anti-inflammatory**	0.285	0.194	0.023	0.13125

^a^ Indicates that the drug appears to be unavailable as an active pharmaceutical agent

^b^ Indicates active as a drug but not suitable for internal use

^c^ Details of the pharmaceutical role of the drugs has come from information obtained from the Inxight web pages [https://drugs.ncats.io/substances]

## Discussion

Our criteria for pursuing a pocket site were that the druggability score should be high, that in the parts of the protein where a three-fold symmetry should be present, the same pocket should be found in each of the subunits, that the site was of a likely suitable volume [11], and that, if possible, the site had a high interest regarding either structure or function within the S protein. The pocket identified in this study is of particular interest as it is located at an interface between two chains, B and C, of the protein and is not present between the comparable residues in A and B or A and C. This is because chains B and C have their mobile domains in the down position while chain A has its mobile domain in the up position. The pocket is between two domains, one flanking region in chain C of the protein (between residues 1 and 290) and the other, the underside of the mobile domain in chain B (Fig. 2). The comparison of the pocket residues of Covid-19 and the matched SARS spike protein residues (Fig. 3) shows a very high degree of conservation between them, adding weight to the suggestion they might be important regarding the function of the protein. The domain in chain B is the region between residues around 335 and 526 in the Covid-19 spike protein structure, but in chain A, this same domain region has hinged away losing all contacts between residues 328 and 530 (in beta strands N- and C-terminal to the domain, respectively) as this domain is in the “up” position. In related spike proteins like that from human SARS-CoV (PDB code: 6ACK), when this domain is in the up position it interacts with ACE2 facilitating entry to the cell [16]. The movement of this domain, therefore, appears to be required for ACE2 interaction, as in the SARS-CoV structure an ACE2 is present and bound to the spike protein. Critically, in the Covid-19 Cryo-EM spike protein structure, when in the up position (and when no ACE2 protein is present) then residues 460 to 473 in chain A are unobserved in the structure; they are too flexible to be detectable (grey coloured structure in Fig. 4b), whereas the equivalent residues are present in the SARS-CoV structure with the ACE2 bound. This implies they are flexible when there is no contact with the ACE2 protein, only being stabilised in this up position once contact has been established. However, residues 464 to 466 are structured and visible in the Covid-19 Cryo-EM B chain and form part of the side of the druggable pocket (Table 1 and Fig. 4) because this chain is in the down position. If the inter-chain interactions within and around this pocket formed between the two domains could be stabilised by the binding in of an appropriate drug molecule, it might prevent the domain from moving to the up position and this could be crucial in preventing or hindering the infectivity of the virus. The location of this pocket near such a functionally important domain, and its high predicted druggability score were the reasons behind this site being chosen for the virtual screening studies.

The averaged pairwise root mean square deviation (avRMSD) calculations between each of the docking methods were used as a guide to the quality of each drug pose being in a well-established position within the druggable pocket. A small RMSD value indicated that each method had placed the specific drug pose into a similar position within the pocket. In vina for example, due to the non-deterministic nature of the docking algorithm [12], both it and Smina [13] use a random seed approach to initiate the search process. The results of the poses from all three methods would ideally produce “comparable” but not “identical” results. For all three methods the poses obtained retain information of rotamer space, and overall structural space, which can be employed to find good candidates for fitting the pocket, but from slightly different start positions. So it would be expected and anticipated that, for a given drug molecule, good poses would be comparable, but not the same, from each of the methods used, which would be reflected in small and similar RMSD pairwise values being obtained. However, given that the scoring of the two methods, vina and Smina, was “expected” to be different, but in fact proved to be highly correlated in their results, we removed the Smina scoring from the overall ComboPC equation as we did not want to bias in favour of these two methods over all three.

For the top 150 poses ranked by ComboPC the number of hydrogen bond acceptors was found to be higher than expected (1.16, *p* < 0.001). The number of rings/heterocycles per compound was also significantly higher (1.29, *p* < 0.001). Compounds were also much more likely to contain halogen atoms (1.74, *p* < 0.001), particularly fluorine atoms (2.16, *p* < 0.001). Compounds with SO_2_ groups were also over-represented (2.97, *p* < 0.001), as exemplified by Ladarixin, Diflumidone and Quinethazone. Moieties of this kind are capable of many electrostatic interactions which need not be associated only with hydrogen-bonding. Given the statistical significance associated with the data above it appears that the properties of the pocket, its shape and lining residue composition, have generated a focused list of drugs with notable component properties of their own.

In the top 100 drug poses, of interest there are 20 anti-inflammatory drugs of which 8 are members of the “profen” family, derivatives containing the 2-arylpropionic acid moiety. Many of the highlighted drugs in Table 2 could be capable of successfully binding into this pocket and hence alter the infectivity profile. Clearly, these would need to be examined experimentally because at such a sensitive site in the spike protein there is a possibility of promoting the mobile domain into the up, infective conformation. However, with the fact that these drugs interact with the residues lining this pocket they might strengthen the interaction forces in this region and prevent the mobile domain from moving.

## Conclusions

This study provides a suggested list of pharmaceutical agents, identifying some of them as being from related structure families, that are available on the market and have been sanctioned for use in humans that have been shown to be capable of binding into a druggable pocket in the spike protein of Covid-19. It must be stated that whether they do or do not actually bind in cannot be ratified here, that would have to be determined experimentally. Some might even bind into the site and increase infectivity as a result. However, the aims of this study were to present and show that the members of this list might have the capacity to bind in, thereby providing open suggestions to experimentalists to establish whether many, some, or few, of these agents do actually bind into the spike protein. Equally, it is not possible to state whether these drugs would be in any way efficacious towards suppressing the infectivity of Covid-19 because that is not the remit of this work. Again, that would be for those in the field to establish whether the listed drugs show any effect on reducing virus titre levels, thereby indicating that they are indeed interfering with the infectivity of the virion. The primary aim has been to show that pharmaceutical agents already available and approved might be usable as drugs to interfere with the process of infection, thereby providing time for the host generation of antibodies to combat this latest of cross-species coronavirus events.

## Methods

### Pocket identification

We utilised the Cryo-EM spike protein structure (PDB code: 6VSB), as a source for our studies to locate the presence of “druggable” pockets where small molecules could bind. The protein atomic coordinates were uploaded to the DoGSiteScorer server [11] to identify potential pockets within the structure. The server provides a druggability score that ranges from 0 to 1, 1 being the most druggable.

### Docking methods

Three approaches to docking the pharmaceutical agents into the identified druggable pocket in the Covid-19 spike protein were employed to act to corroborate the output produced. These were Autodock vina [12], Smina [13] and Ledock [14] . The centre position of the pocket was calculated and a search grid space, defined by those coordinates ±12 Å in the x, y and z coordinate directions from that centre, was created and supplied to each of the docking methods. By default in each of the packages used, the drugs were free to explore their rotamer space to optimise their binding into the pocket. However, whilst it is customary to allow side chain flexibility in the residues lining the pocket when undertaking in silico docking studies, given that the resolution of the structure used here was only 3.5 Å, no flexible side chains were defined.

The number of poses considered by all three methods used was left as default because this was thought to be optimal for this study. For Ledock, a maximum of 20 docking poses was returned per conformer, with an additional pose root mean square deviation (RMSD) cutoff of 1.0 Å applied to reduce redundancy of returned poses. For Autodock vina and Smina, a maximum of 9 poses with a maximum energy range of 3 kcal/mol between best and worst were returned.

A 1049-member library of compounds was derived from the Drugs-lib dataset available from the virtual screening webserver MTIOpenScreen [17] from an initial study we undertook on the S protein model from I-Tasser. This dataset consists of approved drugs and research chemicals refined from the “drug” subset of the ChEMBL database [18], the “approved” subset of DrugBank version 5.0.10 [19], the DrugCentral online compendium [20] and the “approved” SuperDrug2 database version 2.0 [21].

Initially Autodock vina and Ledock were the methods of choice as these have been considered as the best non^_^commercial docking packages available [22]. However, after pairing the poses between the two methods according to their structural similarity when in the docking site (as in the RMSD Calculations below) the corresponding docking scores showed only a weak correlation (0.36, *p* < 0.0001)) and so Smina, which is reported as having a different scoring scheme from Autodock vina [13], was used to see if this improved the correlation of the docking scoring to Ledock. In fact, the scoring correlation between Smina and Autodock vina proved to be very high and so only Autodock vina and Ledock data were used in the docking scoring as a result.

### RMSD calculations

For a given drug, poses were obtained that fit the pocket. To establish the best superposition of these different poses from each method, firstly, pairwise RMSDs were calculated using the following equation:

$$ RMSD=\sqrt{\frac{1}{n}\sum \limits_{i=1}^n{\left({x}_i-{y}_i\right)}^2} $$

where x_i_ and y_i_ are comparable atoms from two of the methods, and n is the number of atoms in the given drug. The best overall superposition from the three methods was then calculated by taking the average of these pairwise RMSD superpositions. From these calculations, the poses from each of the methods with the best overall superposition, and hence, highest degree of similarity, would have the lowest average RMSD values (avRMSD).

### Overall druggability analysis and ranking

An extensive amount of data was generated tabulating the output information from the docking packages of the druggable compounds with their structural characteristics. In generalised overview these correspond to the docking output scores from each of the methods, avRMSD values between the poses chosen for each compound, hydrogen bonding data, and the JOELib Native descriptor set, calculated using the ChemMine webserver [23], which include molar refractivity, polar surface area, and frequencies of atom and selected group types among other geometric and chemical properties. Drugs within the search group were also classified by chemical compound class and, where possible, by subclass through classification using the ClassyFire webserver [24].

A compound with a pose that scored highly in the two scoring regimes, and with good superposition agreement between docking methods, as determined from their avRMSD values, was considered more likely to be a meaningful data point. As these individual terms were on different scales, their significant feature was their explicit “order” rather than “value”. As a result each component, scoring value, and avRMSD, was ranked according to their percentile position within their individual scales. This therefore ordered the results by position rather than by value. Thus, the following combined score, ComboPC, was created to rank the compounds applying equal weight to the estimated docking scores from each docking method and to the agreement between the superpositions from each of the methods, as measured by avRMSD:

$$ ComboPC=\frac{1}{2}\left(\frac{\left( LedockPC+ vinaPC\right)}{2}+ RMSDPC\right) $$

Where LedockPC is the percentile rank of the docking score of the pose as docked by Ledock, vinaPC is the percentile rank of the docking score of the pose as docked by vina, and RMSDPC is the percentile rank of the avRMSD between the poses from each docking method. Only the scores from Ledock and vina, but not Smina, were chosen for inclusion in the ComboPC score as the scores from vina and Smina were found to be highly correlated (0.97, *p* < 0.001)).

## Supplementary information

**Additional file 1.** Supplementary_data_drug_scores.xlsx - The spreadsheet of data used and analysed in this paper.

**Additional file 2.** Binding_pocket_residues.pdb - The binding pocket residues found in this study from the Covid-19 spike protein structure (PDB code: 6VSB).

**Additional file 3.** LEDOCK_top100.mol2 - The top 100 Ledock poses listed in Table 2.

**Additional file 4.** SMINA_top100.mol2 - The top 100 Smina poses listed in Table 2.

**Additional file 5.** VINA_top100.mol2 - The top 100 Autodock vina poses listed in Table 2.

**Additional file 6.** readme.txt - A readme.txt file to provide information to assist in using the structure data provided in Additional files 2-5.

## Data Availability

An Excel spreadsheet of the data obtained in this study and the poses produced from the docking studies are available online as Supplementary Information. These are Additional file 1, Additional file 2, Additional file 3, Additional file 4, Additional file 5 and Additional file 6.

## Abbreviations

ACE2: Angiotensin Converting Enzyme 2
Cryo-EM: Cryo Electron Microscopy
MERS: Middle Eastern Respiratory Syndrome
PDB: Protein Data Bank
RMSD: Root Mean Square Deviation
SARS: Severe Acute Respiratory Syndrome
